# Evaluation of a new disposable silicon limbal relaxing incision knife by experienced users

**DOI:** 10.1186/1471-2415-9-15

**Published:** 2009-12-21

**Authors:** John Albanese, Geoffrey Dugue, Valentin Parvu, Ann M Bajart, Edwin Lee

**Affiliations:** 1 Becton Dickinson and Company, 1 Becton Drive, Franklin Lakes, NJ, USA; 2 Ophthalmic Consultants of Boston, Boston, MA, USA

## Abstract

**Background:**

Previous research has suggested that the silicon BD Atomic Edge™ knife has superior performance characteristics when compared to a metal knife and performance similar to diamond knife when making various incisions. This study was designed to determine whether a silicon accurate depth knife has equivalent performance characteristics when compared to a diamond limbal relaxing incision (LRI) knife and superior performance characteristics when compared to a steel accurate depth knife when creating limbal relaxing incision.

**Methods:**

Sixty-five ophthalmic surgeons with limbal relaxing incision experience created limbal relaxing incisions in *ex-vivo *porcine eyes with silicon and steel accurate depth knives and diamond LRI knives. The ophthalmic surgeons rated multiple performance characteristics of the knives on Visual Analog Scales.

**Results:**

The observed differences between the silicon knife and diamond knife were found to be insignificant. The mean ratio between the performance of the silicon knife and the diamond knife was shown to be greater than 90% (with 95% confidence). The silicon knife's mean performance was significantly higher than the performance of the steel knife for all characteristics. (*p*-value < .05)

**Conclusions:**

For experienced users, the silicon accurate depth knife was found to be equivalent in performance to the diamond LRI knife and superior to the steel accurate depth knife when making limbal relaxing incisions in *ex vivo *porcine eyes. Disposable silicon LRI knives may be an alternative to diamond LRI knives.

## Background

Refractive outcomes have become an increasingly important part of cataract surgery and the limbal relaxing incision (LRI) has been shown to be a safe and effective procedure to reduce astigmatism [1-4]. Currently, ophthalmic surgeons have only two material choices for knives to perform limbal relaxing incision, diamond and metal. BD (Becton, Dickinson and Company), Franklin Lakes, NJ has developed a safety engineered, single use, uni-directional cutting silicon knife for these incisions. Previous research has suggested that the silicon BD Atomic Edge™ knife has superior performance characteristics when compared to a metal knife and performance similar to diamond knife when making various incisions [5,6]. This study was designed to determine the performance characteristics of silicon accurate depth knives in comparison to diamond LRI knives and steel accurate depth knives in five performance characteristics.

## Methods

### Materials

**Figure 1:**
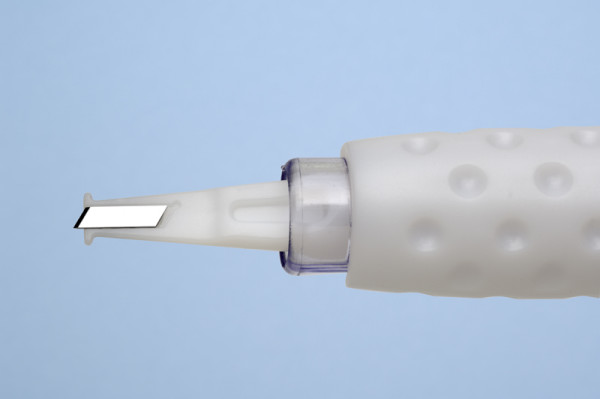
**BD Atomic Edge™ Accurate Depth knife**.

**Figure 2:**
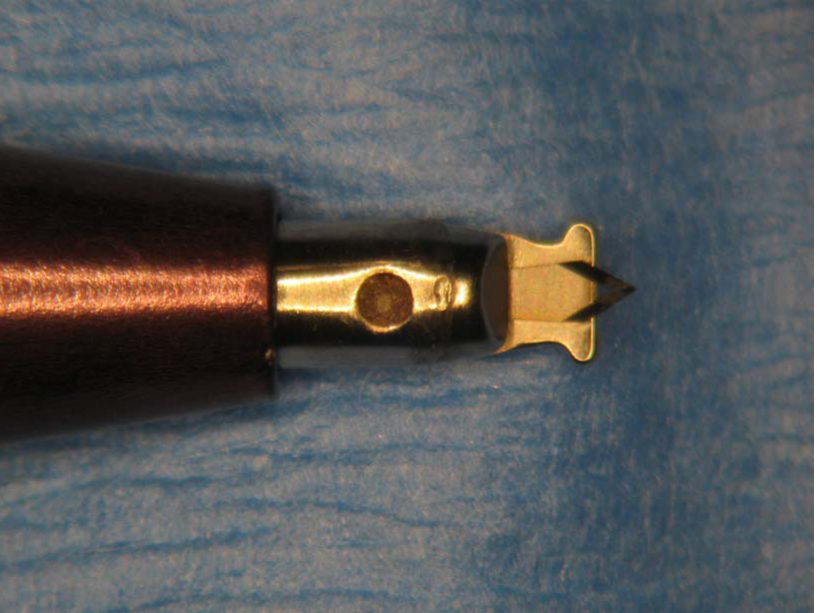
**Accutome Rubenstein LRI knife**.

**Figure 3:**
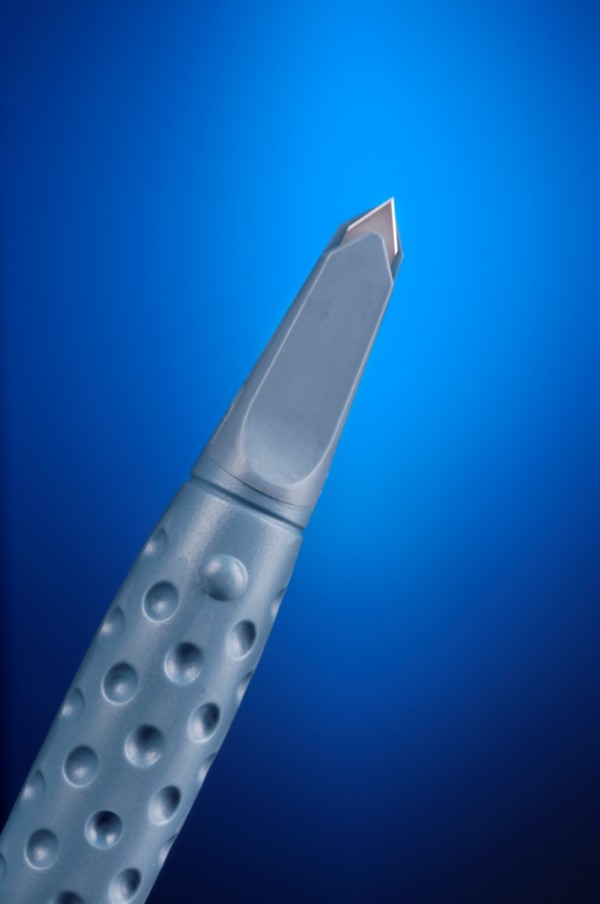
**BD Beaver™ Accurate Depth knife**.

The following six types of accurate depth knives were evaluated: silicon accurate depth knife (BD Atomic Edge™ Accurate Depth knife (figure 1)), diamond LRI knife (Accutome Rubenstein LRI knife (figure 2)), steel accurate depth knife (BD Beaver™ Accurate Depth knife (figure 3)), dulled silicon accurate depth knife (BD Atomic Edge™ Accurate Depth knife), dulled diamond LRI knife (Accutome Rubenstein LRI knife) and dulled steel accurate depth knife (BD Beaver™ Accurate Depth knife). All knives were pre-set at a 600 micron depth. The silicon accurate depth knife is a single, uni-directional cutting knife with the depth preset by the manufacturer. The knife also has a retractable shield to protect the user and the blade, and the shield's slider mechanism is designed to open and close with a single-hand operation. The steel accurate depth knife has the depth also preset by the manufacturer but has bi-directional cutting capabilities. The diamond LRI knife is retractable and can be pre-set to various depths. It is a reusable knife with bi-directional cutting capabilities. Of the three knives the diamond knife is the only one designed for re-use.

The dulled steel and silicon knives were dulled by cutting a piece polyethylene sheet stock with the knives (5 cm length cut for steel and 10 cm length cut for silicon). The dulled diamond knife was dulled by cutting a silicon carbide coarse abrasive pad for a length of 4 cm. All cutting edges were dulled on these knives.

### Methods

Sixty-five ophthalmic surgeons with experience making limbal relaxing incisions were recruited during the American Academy of Ophthalmology 2007 Annual Meeting to evaluate the six different types of knives designed to create limbal relaxing incisions in *ex vivo *porcine eyes. Each surgeon made eight incisions with silicon accurate depth knives, eight incisions with diamond LRI knives and two incisions with steel accurate depth knives, in randomized order. Further, each ophthalmic surgeon evaluated one dulled knife of each type. The silicon and steel knives were disposed of after one use. The diamond knives were cleaned and reused during the study.

Each ophthalmic surgeon rated the following characteristics for each knife after each incision on a Visual Analog Scale (VAS):

a) The smoothness of making the incision

b) How well the curvature of the eye was tracked

c) The control of the incision

d) The overall incision quality

e) The sharpness of the blade

Each evaluation was scored by placing a vertical line on a 150 mm Visual Analog Scale based on where they felt the characteristics of the knife being assessed fell. The Visual Analog Scales in this study were labelled using a negative descriptor on one end and a positive one on the other. For example, the sharpness scale's descriptors were 'Not Sharp' on the left end and 'Exceptionally Sharp' on the right end.

### Statistical Methods

Prior to the study it was expected that differences between the steel knife's performance and the silicon and diamond knives' performance would be larger than differences between the silicon knife's and the diamond knife's performance. For that reason it was decided to have the surgeons perform more incisions with the diamond and silicon knives than with the steel knife. Based on an assumed Coefficient of Variation of 30% (as observed in a pilot study), if 60 surgeons finished this study, then 480 incisions would have been performed with the diamond knife, 480 incisions with the silicon knife, and 120 incisions with the steel knife. This would have allowed the study to have a 81.3% power to conclude non-inferiority if the silicon knife's performance was no more than 5% worse than the diamond knife's performance and a 86.6% power to conclude superiority if the silicon knife's performance was at least 10% better than the steel knife's performance in the characteristics measured.

The VAS measurements of the non-dulled knife ratings were log-transformed and a general linear model with surgeon as a random effect was fitted for each characteristic. Pairwise comparisons using Tukey's adjustment for multiple comparisons were made among the three different knife types.

## Results

Prior to the start of study it was decided to use a non-inferiority margin of 10% to establish equivalence of the silicon knife to the diamond knife with respect to the rated characteristics. To establish superiority over the steel knife it was decided that a greater than 10% performance increase must be observed.

Evaluations from 64 ophthalmic surgeons each making 18 incisions were analyzed. The ophthalmic surgeons that participated in this investigation were predominantly right handed (86.2%) and had a cataract specialty (73.8%). The surgeons had an average of 15.4 years of experience (SD = 9.04 years) and completed an average of 28.9 LRIs in the 6 months prior to the study (SD = 41.6). Surgeons who most often used diamond knives for LRIs accounted for 55.4% of the surgeons. Surgeons who most often used metal knives for LRIs accounted for 44.6% of the surgeons.

**Figure 4:**
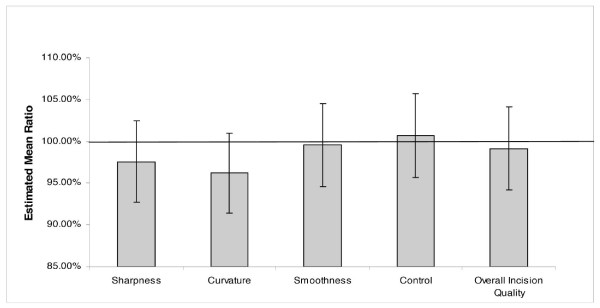
**Silicon versus Diamond**. The estimated mean ratios and confidence intervals for each characteristic assessed for the silicon and diamond knife comparison.

**Figure 5:**
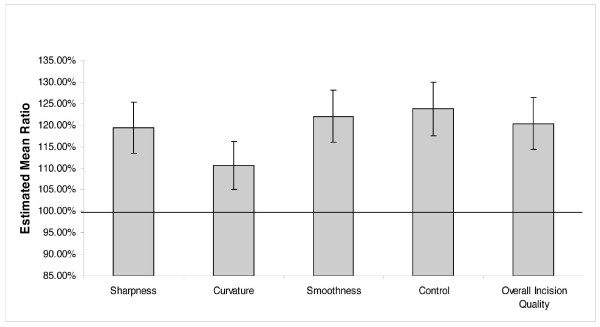
**Silicon versus Steel**. The estimated mean ratios and confidence intervals for each characteristic assessed for the silicon and steel knife comparison.

Tables 1 and 2 display the summary statistics of the Visual Analog Scale ratings for each characteristic evaluated. Table 3 gives the estimated mean ratios for each pairwise comparison along with 95% confidence intervals and p-values for each comparison. Figures 4 and 5 display the estimated mean ratios and 95% confidence intervals for silicon versus diamond and silicon versus steel comparisons.

**Table 1:** Summary statistics for VAS evaluations for the three different blade types

	Blade	N	Mean	SD	CV	Min	Median	Max
Sharpness	Atomic Edge	512	122.9	26.9	21.9	3.6	132.1	146.4
	Diamond	511	123.5	26.4	21.4	3.6	132.1	146.4
	Metal	128	104.6	32.6	31.1	10.7	110.7	146.4
Curvature	Atomic Edge	512	118.5	31.2	26.3	3.6	125.0	146.4
	Diamond	510	118.2	27.8	23.5	3.6	125.0	146.4
	Metal	128	105.6	33.2	31.5	10.7	110.7	146.4
Smoothness	Atomic Edge	512	119.9	29.4	24.5	3.6	128.6	146.4
	Diamond	511	118.0	28.7	24.4	3.6	125.0	146.4
	Metal	128	101.1	35.9	35.6	3.6	110.7	146.4
Control	Atomic Edge	512	118.5	29.2	24.7	3.6	125.0	146.4
	Diamond	511	115.1	28.0	24.3	3.6	125.0	146.4
	Metal	128	101.7	36.2	35.6	3.6	110.7	146.4
Overall	Atomic Edge	510	120.2	28.3	23.6	3.6	132.1	146.4
	Diamond	511	117.7	26.2	22.2	10.7	125.0	146.4
	Metal	128	102.3	34.9	34.1	10.7	110.7	146.4

**Table 2:** Summary statistics for VAS evaluations for dull blades

	Dulled Blade	N	Mean	SD	CV	Min	Median	Max
Sharpness	Atomic Edge	64	116.9	30.2	25.9	3.6	125.0	146.4
	Diamond	64	26.9	31.5	117.1	3.6	10.7	132.1
	Metal	64	66.2	39.8	60.2	3.6	60.7	146.4
Curvature	Atomic Edge	64	113.2	32.3	28.6	3.6	125.0	146.4
	Diamond	64	31.5	35.5	112.9	3.6	10.7	132.1
	Metal	64	74.3	38.0	51.1	3.6	78.6	146.4
Smoothness	Atomic Edge	64	115.2	31.0	26.9	3.6	125.0	146.4
	Diamond	64	24.2	29.6	122.4	3.6	10.7	139.3
	Metal	64	63.3	40.8	64.4	3.6	60.7	146.4
Control	Atomic Edge	64	108.0	37.0	34.3	3.6	125.0	146.4
	Diamond	64	25.6	30.6	119.6	3.6	10.7	139.3
	Metal	64	75.1	37.6	50.0	10.7	78.6	146.4
Overall	Atomic Edge	64	113.2	31.8	28.1	3.6	125.0	146.4
	Diamond	64	25.6	31.1	121.8	3.6	10.7	139.3
	Metal	64	68.4	39.1	57.1	10.7	67.9	139.3

**Table 3:** Pairwise comparisons among the three different blade types.

	Blade Comparison	Estimated Mean Ratio (95% Confidence Interval)	Adjusted *p*-value
Sharpness	Atomic Edge/Diamond	97.55% (92.57%, 102.8%)	0.507
	Atomic Edge/Metal	119.4% (109.91%, 129.7%)	<.0001
	Diamond/Metal	122.4% (112.67%, 132.96%)	<.0001
Curvature	Atomic Edge/Diamond	96.2% (90.4%, 102.38%)	0.3104
	Atomic Edge/Metal	110.61% (100.26%, 122.02%)	0.0426
	Diamond/Metal	114.97% (104.22%, 126.84%)	0.0026
Smoothness	Atomic Edge/Diamond	99.58% (93.38%, 106.19%)	0.987
	Atomic Edge/Metal	122.06% (110.27%, 135.12%)	<.0001
	Diamond/Metal	122.58% (110.73%, 135.69%)	<.0001
Control	Atomic Edge/Diamond	100.7% (94.5%, 107.31%)	0.9639
	Atomic Edge/Metal	123.77% (111.95%, 136.85%)	<.0001
	Diamond/Metal	122.91% (111.16%, 135.89%)	<.0001
Overall	Atomic Edge/Diamond	99.15% (93.83%, 104.77%)	0.9297
	Atomic Edge/Metal	120.4% (110.37%, 131.35%)	<.0001
	Diamond/Metal	121.44% (111.32%, 132.48%)	<.0001

Statistically, the observed differences between the silicon knives and diamond knives were insignificant (p-value > .05) and the lower 95% confidence limit for the mean ratio was greater than 90% for all performance measurements. The silicon knife mean performance was statistically significantly higher than the mean performance of the steel knife for all characteristics (p-value < .05). The improvement in performance ranged from 11% to 24% depending on the specific characteristics. Similarly, the performance of the diamond knife was also found to be significantly higher than the performance of the steel knife (p-value < .05).

## Discussion

Currently only metal accurate depth knives and diamond LRI knives are available to ophthalmic surgeons for the creation of limbal relaxing incisions. While diamond knives are generally felt to be superior in performance, their cost may be prohibitive in a resource constrained surgical environment, and risk of contamination of instruments by prions or other agents between uses has been of concern [6-9].

In this study, all participating ophthalmic surgeons were experienced and it is likely that many had a pre-existing preference for one of the currently available accurate depth knife types. To overcome this potential bias, dulled knives of each type were introduced into the testing sequence. The ophthalmic surgeons were not made aware which specific knives were dulled but they were informed that some knives that they were to evaluate had been dulled. This was done in order to provide the surgeon with the expectation of using knives in the study that behaved significantly differently from their prior experience with similar knives, so as to heighten their awareness of potentially subtle differences among the knives tested. The likely success of this technique may be appreciated by the clustering of ratings for the dulled knives significantly below the steel version of the sharp knives. Clearly surgeons were judging what they felt with their hands and not what they expected based on visual identification of the knife types.

As previously stated, *ex vivo *porcine eyes were used as the model for the evaluation of the performance of the knives. Prior to the execution of this study, a laboratory evaluation was conducted to ensure using eyes of one, two and three days from harvest would not introduce any bias into the study. An ophthalmic surgeon* with many years of experience performing LRIs was asked to evaluate the amount of epithelial damage after each incision. It was found that epithelial damage did increase with the post harvest age of the eyes. However, the difference between the amount of damage caused by the incisions by each knife, did not change based on the post-harvest age of the eyes. Based on this information it was decided that each ophthalmic surgeon in the knife evaluation study would create their eighteen incisions in porcine eyes of the same post-harvest age to avoid any bias.

To further limit any potential bias in this study, a secluded booth at the far edge of the New Orleans Convention Center exhibit hall was used, completely independent of the commercial BD booth. The physical distance aided in ensuring that the study participants consisted of a wide variety of surgeons who visited the trade show floor rather than merely those who wished to visit the BD booth. The study was conducted by the BD Clinical Trial Resources Department and all study conduct was monitored under strict GCP (Good Clinical Practices) guidelines. The study staff was trained to not discuss design of the devices being evaluated or any topics related to BD or BD products in order to prevent their opinions from influencing the surgeon's ratings. Only study staff and the participating ophthalmic surgeons were allowed into the booth to limit any influence that sales or marketing personnel may have had on the evaluations. The study population was also representative of those who would use the products for surgery. As displayed in the study results, the surgeons who participated in this trial had an average of 15.4 years of experience, an average frequency of 28.9 limbal relaxing incisions performed in the 6 months prior to the study and a roughly even division of diamond/steel knife users. Also, all Visual Analog Scale measurements were made by an optical scanner to reduce measurement error and bias. The order of the evaluations was also randomized in an effort to eliminate any bias that could have occurred due to rating the knives in a consistent order. Randomization also should have reduced the effect of any unknown variables on the outcome measures.

Even though this study was designed to overcome many areas that may have introduced bias, the study is not without limitations. First, the experience of performing an incision in the e*x-vivo *porcine eye may not be representative of an incision made in a human eye. The difference between live human eyes and an *ex-vivo *porcine may impact a surgeon's perception of a device. However, since each device was compared in the same model, these relative performance assessments should hold true in most applications. Further, porcine eyes have been shown to provide reliable data analogous to that from humans in many areas of Ophthalmology [10-12]. As described, steps were taken to standardize the model through controlling the age of the eyes following harvest. Eye pressure and eye moistness were also maintained in a standardized fashion across all eyes. Eye pressure control was provided by the use of suction on the back of the porcine eye to simulate intraocular pressure. The same stand also provided stabile fixation of the eye. Appropriate moisture on the cornea was maintained by frequent spraying of balanced salt solution on the eyes throughout the evaluations. While no model can fully represent the condition of surgery on living, human tissue, our intent was to provide the most accurate model possible.

We could not assess whether important clinical outcomes such as wound healing and wound stability would be improved by the use of instruments surgeons perceive to have better performance characteristics, such as being sharper. However, to the extent that sharpness may be correlated with better operator performance, we believe that the clinical outcomes found when using the silicon knife would be equivalent to that of the diamond knife. Furthermore, one may consider that patient safety may well be improved with the decreased risk of patient to patient contamination resulting from utilization of a single use device.

There is currently no 'gold standard' objective definition of sharpness. Therefore, we decided that an evaluation of the subjective experience of highly qualified users would be the best way to assess the differences between these devices. There is also no accepted scale or standard descriptors to measure sharpness (or any of the characteristics assessed), hence we believe the descriptive terms we used, validated as they were within the study, are the best tool we have to capture human perception of the sharpness of these devices. Even though precise measurements may be performed in a laboratory setting there is not sufficient research to link measurements (such as drag for or blade architecture) to a conclusion of which device surgeons would prefer. The descriptors we used in a standard VAS format were able to repeatably discriminate differences between test groups in a predictable, logical direction. For example, scores on the scales for diamond knives that were purposely dulled were found to be grouped near each other and these values were significantly lower than scores for identical knives which were not dulled. Overall, even though this study does not answer all the questions in regard to which knife to choose when making a limbal relaxing incision, it does provide a reasonable approach for future evaluations of LRI knives.

## Conclusions

This study has demonstrated that the silicon accurate depth knife is equivalent to the diamond accurate depth knife and both are superior to the steel accurate depth knife in each characteristic assessed. The combination of these characteristics suggests that the silicon knife should perform as well as a diamond knife and better than a steel knife in making limbal relaxing incisions.

* Ann M. Bajart, MD, F.A.C.S

## Abbreviations

BD: Becton Dickinson and Company; GCP: Good Clinical Practice; LRI: Limbal Relaxing Incision; SD: Standard Deviation; VAS: Visual Analog Scale

## Competing interests

Conflicts of Interest: All authors are either employed by or consult for BD, which manufactures the silicon and steel knives used in this study.

## Authors' contributions

JA participated in the design of the study and drafted the manuscript. GD conceived the study, participated in the design of the study and helped draft the manuscript. VP participated in the design of the study and performed the statistical analysis. AMB performed the aged porcine eye evaluation. EL participated in the design of the study and analyzed the aged porcine eye data. All authors read and approved the final manuscript.

## Pre-publication history

The pre-publication history for this paper can be accessed here:

http://www.biomedcentral.com/1471-2415/9/15/prepub
